# Potential Role of Olive Oil Phenolic Compounds in the Prevention of Neurodegenerative Diseases

**DOI:** 10.3390/molecules20034655

**Published:** 2015-03-13

**Authors:** Jose Rodríguez-Morató, Laura Xicota, Montse Fitó, Magí Farré, Mara Dierssen, Rafael de la Torre

**Affiliations:** 1 Human Pharmacology and Clinical Neurosciences Research Group, Neurosciences Research Program, IMIM-Institut Hospital del Mar d’Investigacions Mèdiques, Dr. Aiguader 88, Barcelona 08003, Spain; E-Mails: jrodriguez1@imim.es (J.R.-M.); lxicota@imim.es (L.X.); mfarre@imim.es (M.F.); 2 Department of Experimental and Health Sciences, Universitat Pompeu Fabra (CEXS-UPF), Dr. Aiguader 80, Barcelona 08003, Spain; 3 CIBER de Fisiopatología de la Obesidad y Nutrición (CIBEROBN, CB06/03/028), Santiago de Compostela 15706, Spain; E-Mail: mfito@imim.es; 4 Cellular & Systems Neurobiology Research Group, Center of Genomic Regulation, Dr. Aiguader 88, Barcelona 08003, Spain; E-Mail: mara.dierssen@crg.eu; 5 Cardiovascular Risk and Nutrition Research Group, Epidemiology Program, IMIM-Institut Hospital del Mar d’Investigacions Mèdiques, Dr. Aiguader 88, Barcelona 08003, Spain; 6 Department of Pharmacology, Therapeutics and Toxicology, Universitat Autònoma de Barcelona, Barcelona 08193, Spain; 7 CIBER de Enfermedades Raras (CIBERER), Barcelona 08003, Spain

**Keywords:** olive oil, phenolic compounds, hydroxytyrosol, tyrosol, neurodegenerative diseases, prevention, neuroprotective agents

## Abstract

Adherence to the Mediterranean Diet (MD) has been associated with a reduced incidence of neurodegenerative diseases and better cognitive performance. Virgin olive oil, the main source of lipids in the MD, is rich in minor phenolic components, particularly hydroxytyrosol (HT). HT potent antioxidant and anti-inflammatory actions have attracted researchers’ attention and may contribute to neuroprotective effects credited to MD. In this review HT bioavailability and pharmacokinetics are presented prior to discussing health beneficial effects. *In vitro* and *in vivo* neuroprotective effects together with its multiple mechanisms of action are reviewed. Other microconstituents of olive oil are also considered due to their potential neuroprotective effects (oleocanthal, triterpenic acids). Finally, we discuss the potential role of HT as a therapeutic tool in the prevention of neurodegenerative diseases.

## 1. Dietary Phenolic Compounds: Potential Therapeutic Tools in the Prevention of Neurodegenerative Diseases

Polyphenols and simple phenols are secondary metabolites of plants abundant in our diet, including fruits, vegetables, olive oil, wine, and tea. Several studies have indicated that the risk of suffering certain diseases is reduced or increased depending on the food categories consumed. Although this fact has been primarily described in cardiovascular diseases, it has also been related to neurodegenerative disorders [1]. Epidemiological data suggest that certain nutrients (such as antioxidants, E and B-vitamins, and polyunsaturated fatty acids (PUFA) [2]) and foods (such as wine, fish, vegetables, and fruits [3]) may confer protection against cognitive impairment, particularly against Alzheimer’s Disease (AD) [4].

Neurodegenerative diseases tend to present an imbalance between reactive oxygen species production and the antioxidant defense system. The particular evidence of oxidative stress in AD indicates the potential role that antioxidants, such as phenolic compounds from dietary plants, could play in its prevention and management. The natural origin of these phenolic compounds, and their reactivity towards multiple targets, has converted them into potentially useful tools in order to deal with the multifactorial etiology of these diseases [5].

Although dietary phenolic compounds are good antioxidants *in vitro*, *in vivo* such effects may be indirectly mediated through the activation of some pathways (e.g., the Nrf2/Keap1 pathway, discussed in this review) and not by their intrinsic antioxidant activity. In relation to this pathway-modulating ability, there is a newly emerged concept concerning the biological effects of phenolic compounds with respect to their nutrigenomic effects derived from interacting with biological systems [6,7]. Another additional aspect to be considered when attempting to explain dietary phenolic compound biological activity is its ability to alter brain perfusion via its effects on lipid metabolism, thus lowering the risk of stroke [8,9,10].

## 2. The Mediterranean Diet and Cardiovascular and Neurodegenerative Diseases

The term Mediterranean Diet (MD) was coined in the 1960s by Ancel Keys when explaining the results of an epidemiological study in which Italian and Greek populations had lower mortality rates, and a reduced incidence of cancer and cardiovascular disease, compared to other populations [11]. Known as the Seven Countries Study it included more than 12,000 subjects from America, Europe, and Asia. Since then, a new field of clinical and epidemiological research has emerged, and several studies have been carried out aiming at confirming these results, as shown by the exponential increase from 1999 of original articles regarding the MD [12].

The MD is characterized by a high intake of cereals, vegetables, legumes, fruit, and unsaturated fatty acids (mostly from olive oil); low consumption of red meat, poultry products, and saturated fatty acids; and moderate intake of fish, milk and dairy products, as well as a regular but moderate ethanol consumption (generally in the form of wine during meals) [13,14,15,16].

The overall conclusions of several studies concerning the MD confirm that high adherence to this diet reduces the risk of suffering from several pathological conditions including cardiovascular and cerebrovascular diseases, diabetes mellitus, metabolic syndrome, certain cancers, and neurodegenerative diseases [17]. The link between adherence to the MD and a reduction in total mortality has also been confirmed [15,16]. In a prospective cohort study of 1410 elderly French people, a significant association was found between higher adherence to MD and slower cognitive decline [13]. These results were further confirmed in a population of 447 elderly subjects with a high risk of cardiovascular diseases where adherence to MD (using questionnaires and urinary olive oil polyphenols as biomarkers of intervention compliance) was related to better cognitive performance [4]. Recently, further evidence indicates that an intervention with the MD improves cognition [18].

Although the majority of these studies have been carried out in Mediterranean populations, research on the MD in non-Mediterranean countries suggests that health benefits can be transferred to other populations. As an example, a study carried out in more than 2,000 individuals in New York concluded that higher adherence to the MD was associated with a reduction in the risk of AD [14].

## 3. Olive Oil Phenolic Compounds and MD

**Figure 1:**
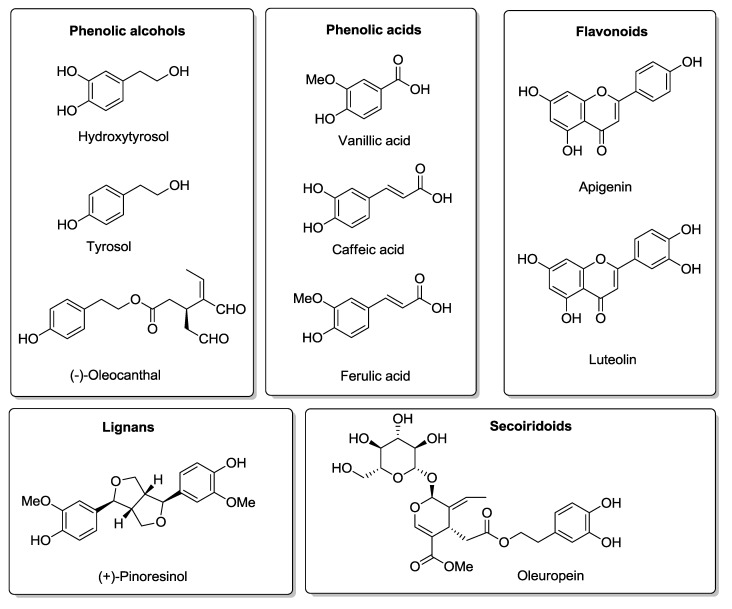
The different classes of polar phenolic compounds present in olive oil with molecular structures of representative examples.

The main source of fat in the MD is olive oil. In Mediterranean populations it is estimated that individuals consume between 25 and 50 mL of olive oil per day (raw olive oil and that used for cooking). Recent cohort studies and randomized clinical trials have provided first level evidence showing that intake of virgin olive oil, rich in phenolic compounds, provides benefits on secondary [19] and primary end points for cardiovascular disease [20]. The health benefits promoted by olive oil cannot only be attributed to its high content in monounsaturated fatty acids (MUFA), mainly oleic acid, but also to its microconstituents, in particular the phenolic compounds [19,21,22]. There are approximately 230 chemical compounds in olive oil including aliphatic and triterpenic alcohols, sterols, hydrocarbons, volatile and phenolic compounds. The main antioxidants of virgin olive oil are carotenes and phenolic compounds including lipophilic and hydrophilic phenols [23]. The polar phenolic compounds that are present in olive oil can be classified as: phenolic alcohols, phenolic acids, flavonoids, lignans, and secoiridoids [24,25,26]. The chemical structures of representative examples of each class are depicted in Figure 1.

**Figure 2:**
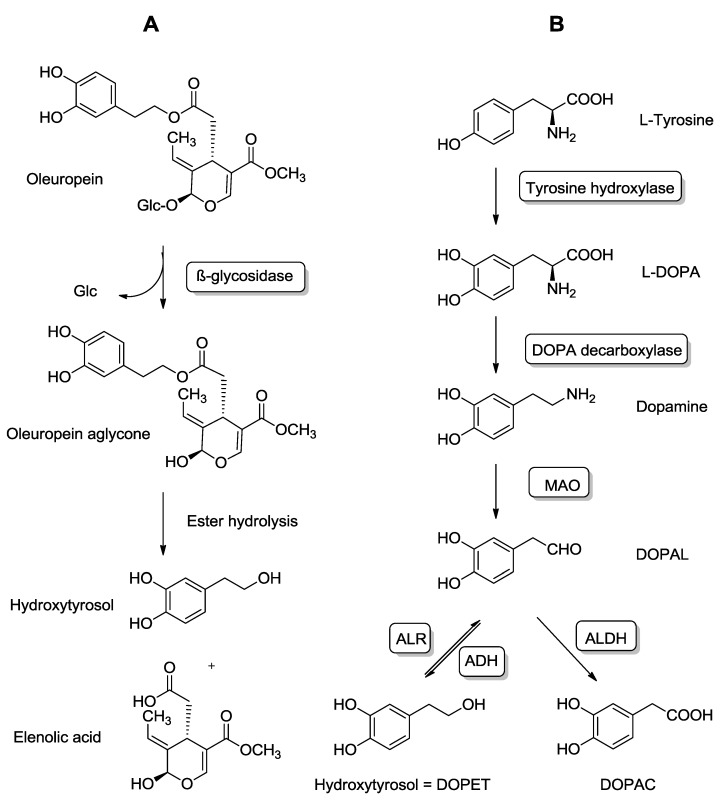
Comparison between exogenous and endogenous sources of hydroytyrosol (HT). (**A**) Origin of HT from oleuropein. Oleuropein hydrolysis results in oleuropein aglycone, whose subsequent hydrolysis originates elenolic acid and HT. (**B**) Endogenous formation of HT via dopamine oxidative metabolism. Abbreviations: ALR: Aldehyde/aldose reductase; ADH: Alcohol dehydrogenase; MAO: Monoamine oxidase; ALDH: Aldehyde dehydrogenase; DOPET: 3,4-dihydroxyphenylethanol; DOPAL: 3,4-dihydroxyphenylacetaldehyde; DOPAC: 3,4-dihydroxyphenylacetic acid; L-DOPA: l-3,4-dihydroxyphenylalanine; Glc: Glucose.

The principal phenolic compounds in olive oil are the secoiridoids, the glycosylated forms of the aglycones oleuropein, and ligstroside [23]. In the gastrointestinal tract, they are mainly hydrolyzed to give rise to hydroxytyrosol (HT) and tyrosol, respectively [27] (Figure 2A). In addition, free HT and tyrosol at very low concentrations in fresh olive oil may increase with olive oil aging. Median concentrations in olive oils may range from 1.9 mg/kg for HT to 163.6 mg/kg for oleuropein [23]. Globally, the amount of polyphenols in olive oil ranges from 200 to 1000 mg/kg depending on the cultivar and agricultural practices. Therefore, the daily dose for HT (combining the volume of olive oil and amount of polyphenol) may not exceed 7 mg/day.

The European Food Safety Authority (EFSA) recently released a health claim concerning phenolic compounds in olive oil and their ability to protect low-density lipoprotein (LDL) from oxidative damage. The minimum daily requirement of HT in the diet was set at 5 mg for these beneficial effects to occur [28]. Since the concentrations of HT in some olive oils may be too low, the EFSA claim could encourage the preparation of HT-enriched olive oils, nutraceuticals, and other food preparations at concentrations much higher than those encountered in its natural form [28].

In relation with this issue, there is currently no official method to quantify all the forms of HT and tyrosol present in olive oil. When comparing different olive oils or nutraceuticals it is important to use appropriate analytical methods that provide an accurate estimation of the olive oil polyphenol content, as considerable variations have been described when employing different methods [29]. Such analytical issues are relevant considering de threshold concentration defined by EFSA. Currently, a number of analytical methods to analyze total levels of HT and tyrosol in olive oil have been developed [30,31,32] and the International Olive Council (IOC) is evaluating them in order to propose an official one to standardize the quantification of olive oil phenols [33].

## 4. Hydroxytyrosol

### 4.1. Exogenous and Endogenous Sources of HT

HT high antioxidant activity has sparked research in several fields, the most noteworthy being the prevention of cardiovascular diseases [34]. Several health promoting properties have been attributed to HT including: antioxidant, cardioprotective, antitumoral, antimicrobial, antidiabetic, and neuroprotective activities [35]. Recent studies have also provided evidence HT-related beneficial effects on the liver, where olive oil phenols inhibit the synthesis of lipids, cholesterol and triglycerides [36,37].

There are two known sources of HT: (i) an exogenous one, which follows the ingestion of natural products that contain HT or its precursors, and (ii) an endogenous one, derived from dopamine oxidative metabolism.

HT is the main phenolic compound found in olives and olive products. A double hydrolysis of oleuropein takes place firstly during olive maturation and storage, and later in the gastrointestinal tract, which gives rise to HT [27,38] (Figure 2A). Although the major dietary source of HT is olive oil, its presence in lower amounts has also been described after red wine ingestion [39].

Additionally, HT can be a byproduct of dopamine oxidative metabolism [39,40]. Indeed, this *ortho*-diphenolic compound can be produced endogenously as it is also a product of dopamine oxidative metabolism known as DOPET (3,4-dihydroxyphenylethanol) [41].

Monoaminooxidase (MAO) catalyzes the oxidative-deamination of dopamine giving rise to the aldehyde metabolite DOPAL (3,4-dihydroxyphenylacetaldehyde), which can then be subsequently oxidized by aldehyde dehydrogenase to the corresponding carboxylic acid (3,4-dihydroxyphenylacetic acid, DOPAC). Although DOPAC is the major metabolite of dopamine in biological matrices, a small portion of DOPAL is reduced to DOPET by aldehyde or aldose reductase [42,43] (Figure 2B). After alcohol intake DOPET may become more relevant quantitatively due to the interaction of ethanol with the dopamine oxidative metabolism [44].

With respect to dopamine metabolism, Parkinson’s disease is associated with an intra-neuronal autotoxic mechanism. It has been postulated that dopamine can act as an endogenous neurotoxin whose toxicity could explain the selective loss of dopaminergic neurons in substantia nigra that characterizes Parkinson’s disease [45]. The highly reactive metabolite DOPAL is even more toxic to dopaminergic cells than dopamine itself, suggesting that DOPAL is the toxic dopamine metabolite *in vivo* [42,46]. Lipid peroxidation processes have been described as generating highly reactive aldehydes as byproducts (including 4-hydroxynonenal and malondialdehyde) which are biomarkers of oxidative stress, as well as inhibitors of aldehyde dehydrogenase 2 (ALDH2). As a consequence of this inhibition, these aldehydes prevent the oxidation of DOPAL to DOPAC, blocking this major metabolic pathway and yielding an accumulation of the neurotoxin DOPAL [47,48]. Taking the previous observations into account, DOPET formation could be used as an endogenous defense mechanism to reduce the levels of DOPAL in dopaminergic neurons by employing a normally minor dopamine metabolic pathway.

This link between oxidative stress and the generation of endogenous neurotoxins opens up a research field focused on the mechanisms involved in neurotoxicity and neurodegeneration which are particularly relevant in the study of Parkinson’s disease pathogenesis.

### 4.2. Pharmacokinetics and Metabolic Disposition of HT

A key requisite to be considered when studying the biological effects that a chemical compound may exert *in vivo* are its pharmacokinetic properties. In this sense, both the bioavailability and the metabolism of HT have been widely researched [49,50,51].

HT is absorbed in a dose-dependent manner from olive oil in humans [52]. After the intake of doses of virgin olive oil that are close to that consumed daily in the MD (25–50 mL/day), HT (and also tyrosol) have been detected and quantified in biological fluids [22,50,53]. The human absorption of high amounts (2.5 mg/kg) of pure HT administered as an aqueous solution was very low and produced a great variety of metabolites, leading to a poor bioavailability (<10%) [54]. This and previous studies [55] evidence the important role that the vehicle of administration plays on HT bioavailability.

The tissue distribution of HT has been studied administrating ^14^C-labelled HT to rats. Notably, according to its potential role as a neuroprotective agent, it is able to cross the blood-brain barrier, although it presents a low brain uptake [56].

**Figure 3:**
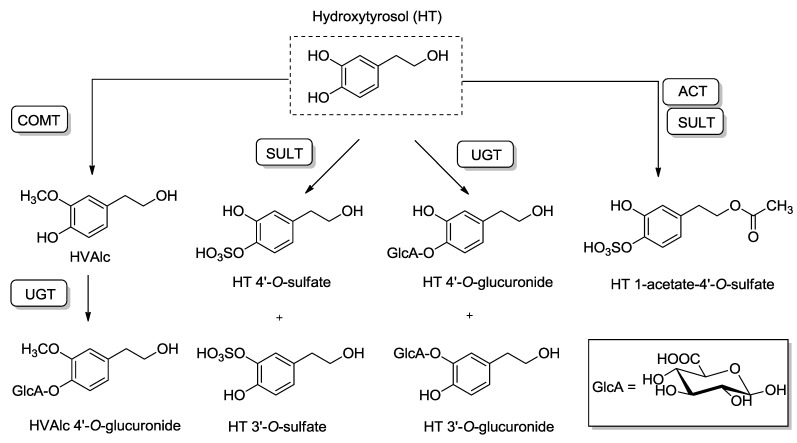
Biotransformation pathways of hydroxytyrosol (HT). Abbreviations: COMT: Catechol-*O*-methyltransferase; UGT: UDP-glucuronosyltransferase; SULT: Sulfotransferase; ACT: *O*-Acetyltransferase; GlcA: Glucuronic acid; HVAlc: Homovanillyl alcohol.

Despite its good absorption, HT bioavailability is poor due to an extensive first pass metabolism. Before entering the portal blood stream, it appears to undergo phase I/II metabolism in the enterocytes, and after having reached the liver through portal circulation, it is subject of additional phase II metabolism. The enzymes implicated in HT phase II metabolism are uridine 5'-diphosphoglucuronosyl transferases (UGTs), catecholmethyltransferase (COMT) [57], and sulfotransferases (SULT) (Figure 3).

The remaining amount of unaltered HT in the bloodstream is extremely low, to such an extent that the free form has been considered undetectable in plasma [39]. Indeed, around 98% of HT and tyrosol in urine and plasma appear to be conjugated as glucuronides and sulfates after olive oil intake [49].

The major metabolic products of HT described in humans are the 3- and 4-*O*-glucuronide conjugates, as well as the corresponding sulfates. COMT catalyzes the conversion of HT into homovanillyl alcohol (HValc) which constitutes a minor metabolic pathway. The free phenolic hydroxyl group of HValc can be also further *O*-glucuronoconjugated [49]. Recently, HT acetate sulfate has also been reported as a biological metabolite of HT [58]. Studies in rats have shown that there are changes in the metabolic disposition of HT in a dose-dependent manner, including the formation of adducts with glutathione (GSH) as measured through the detection of HT mercapturates in urine. Essentially, glucuronoconjugation, the main metabolic pathway at low doses, shifts to sulfation at higher doses. This observation could be of relevance in the context of nutraceuticals prepared at doses higher than those obtained through dietary sources [59].

### 4.3. Antioxidant Properties of HT: From Scavenging Activity to Nrf2 Induction

Two mechanisms have been proposed with respect to HT antioxidant activity: (i) by directly scavenging reactive oxygen species generated during oxidative stress [60], and (ii) by activating different cellular signaling pathways that would increase the organism defenses against an oxidative stress [61]. Also, the interaction of HT with miRNAs, although not yet described, should be considered as a potential molecular target for eliciting its biological effects as described for other polyphenols [62].

Initially, the wide variety of HT biological activities was associated with its strong antioxidant and radical-scavenging activities [60,63]. Phenols have the capacity to donate the hydrogen atom of the phenolic hydroxyl group to free radicals. The presence of a second hydroxyl group at the *ortho*-position enhances antioxidant capacity by generating a catechol ring, which increases the rate at which the hydrogen atom is transferred to peroxyl radicals [64]. Catechols exert a direct antioxidant activity by transforming the catechol moiety into an *ortho*-benzoquinone. Despite containing this catechol moiety and the *in vitro* demonstration of this direct antioxidant capacity, the poor bioavailability of HT in humans practically precludes a direct action of HT on such antioxidant activities, indicating that other mechanisms are involved.

There are data supporting the fact that polyphenols modulate gene expression and that the benefits associated with polyphenol-rich olive oil consumption could be mediated through an *in vivo* nutrigenomic effect in humans [6,7,65]. Additionally, current studies support the concept that HT may confer additional antioxidant protection by increasing the endogenous defense systems. One of the proposed mechanisms recently studied involves the HT-mediated induction of phase II detoxifying enzymes via nuclear factor E2-related factor 2 (Nrf2) activation [61,66,67,68,69,70,71].

The activity of Nrf2 is primarily regulated via its interaction with Kelch-like ECH-associated protein 1 (Keap1) which directs the transcription factor for proteasomal degradation [72,73]. It is generally accepted that modification (e.g., chemical adduction, oxidation, nitrosylation or glutathionylation) of one or more critical cysteine residues in Keap1 represents a likely chemico-biological trigger for the activation of Nrf2 [74]. When Nrf2 escapes Keap1 inhibition it translocates to the nucleus where it interacts with the protein small Maf (sMaf), forming Nrf2–sMaf heterodimers that bind the antioxidant responsive elements (ARE) to regulate the gene expression of several phase II detoxifying enzymes. Nevertheless, there are alternative mechanisms of Nrf2 regulation to be considered, including phosphorylation of Nrf2 by various protein kinases (PKC, PI3K/Akt, GSK-3β, JNK), interaction with other protein partners (p21, caveolin-1), and epigenetic factors (micro-RNAs -144, -28 and -200a, and promoter methylation) activity [75,76,77,78,79].

The first research involving the effects of HT towards the Keap1/Nrf2 pathway was performed by Liu *et al.* who demonstrated that HT could protect mitochondria against acrolein-induced oxidative damage in retinal pigment epithelial cells. In this study, the significant decrease in nuclear Nrf2 expression caused by acrolein could be prevented by HT pre-treatment of the cells [66]. The same group later reported that HT activated Nrf2 to promote the expression of phase II enzymes (including GCL, NQO1, HO-1), and stimulated mitochondrial biogenesis via activation of peroxisome proliferator-activated receptor gamma, co-activator 1 alpha (PPARGC1α) [67].

A further step in the research of the underlying mechanism by which HT induces Nrf2 nuclear translocation has been carried out using specific kinase inhibitors [61,68,69]. HT attenuated *tert-*butyl hydroperoxide-induced damage in HepG2 cells by increasing the activity of three GSH-related enzymes (GSH peroxidase, GSH reductase, and GSH S-transferase). HT also induced Nrf2 nuclear translocation and increased Akt and ERK1/2 phosphorylation. The use of LY294002 and PD98059 (specific inhibitors for the PI3K and ERKs, respectively) decreased Nrf2 nuclear levels, and suppressed the increased mRNA expression, protein expression and activities of the GSH-related enzymes. These results confirmed that PI3K/Akt and ERK1/2 pathways are involved in HT-dependent induction of antioxidant enzymes [68]. Similar results were obtained by Zrelli *et al.*, using vascular endothelial cells. In this study, HT increased cell-proliferation and conferred protection against H_2_O_2_ oxidative stress-induced damage by the induction of heme oxygenase-1 (HO-1) [69].

Zou *et al.* found that HT induced phase II enzymes (HO-1, NQO-1, GCL) and GSH activated the PI3K/Akt pathway in human retinal pigment epithelial cells. However, differing from the two previously mentioned studies, HT effects were not repressed by the PI3K inhibitor LY294002. The authors suggested that c-Jun N-terminal Kinase (JNK) activation was involved in HT-mediated Nrf2 activation [61]. A recent contribution to the better understanding of the HT mechanism of action has been reported by Sgarbossa *et al.* whose research with human keratinocyte cells concluded that HT increases nuclear factor levels of Nrf2 and decreases nuclear levels of Bach1 (a transcription factor that under normal circumstances represses the ARE sequences) [70].

The Nrf2/ARE is emerging as a key neuroprotective pathway in neurodegenerative diseases and its pharmacological activation may represent a neuroprotective therapy [80]. These previously mentioned studies whilst of interest have the limitation of being *in vitro*. However, to evidence the practical relevance of the mechanism, *in vivo* studies aiming to demonstrate that Nrf2 is activated and confers protection are needed.

The senescence-accelerated mouse P8 (SAMP8) is a model of early learning and memory decline. The brains of the SAMP8 overproduce amyloid precursor protein (APP) amyloid-beta peptides and have an increased phosphorylation of tau [81,82]. In fact, this model has been proposed in the development of therapies for AD [83]. Recently, the olive oil-mediated induction of Nrf2 has been demonstrated in 9-week-old SAMP8 mice. In this experiment, two groups of 11 female mice were fed during 4.5 months with semisynthetic diets containing either high or low amounts of olive oil phenolic compounds. Mice fed with the high amount of phenolic compounds presented lower concentrations of oxidative stress markers in the heart tissue, higher levels of Nrf2 mRNA, and higher expression of the antioxidant enzymes GST, GCL, NQO-1, and PON. Additional cell culture studies demonstrated that HT was the only olive oil phenol able to increase Nrf2 transactivation which suggested that this polyphenol may be responsible for the induction of Nrf2-dependent gene expression [71]. Although cognitive effects in this animal model were not investigated, results provide potential for further research into the interaction of HT, Nfr2, and cognitive decline.

Globally, the previously mentioned studies indicate that HT activates Nfr2 in different tissues, and preliminary results *in vivo* suggest that through this activation beneficial effects in neurodegenerative diseases may be elicited.

### 4.4. HT Neuroprotective Properties: In Vitro Evidence

#### 4.4.1. Direct Antioxidant Actions

The neuroprotective effects of HT have been principally studied using two different strategies; the first employs chemically induced neurotoxicity (via oxidative or nitrosative stress) whereas the second is based on the biochemical alterations that take place during the process of hypoxia-reoxygenation. In Table 1, there is a summary of the main *in vitro* and *ex vivo* studies related to HT neuroprotective effects in which this compound acts through a direct antioxidant action.

Hashimoto *et al.* exposed dopaminergic neurons (differentiated PC12 cells) to H_2_O_2_ in the presence or absence of Fe^2+^ in order to study the role of HT in oxidative stress-induced cell damage. As a consequence of this induced oxidative-stress, there was a dose-dependent leakage of lactate dehydrogenase and a decrease in cell viability. The pre-treatment of these cells with HT exerted a protective effect that was attributed to the augmented activity of catalase while GSH levels and GSH peroxidase activity did not change. The molecular mechanisms underlying this increase in catalase activity were not, however, elucidated [84]. Studies with primary cell cultures provided more insight. Koo *et al.* demonstrated that HT significantly protected primary cultures of rat cortical cells from glutamate-induced toxicity. HT was able to attenuate a great variety of toxicological effects caused by high (100 µM) amounts of this excitatory neurotransmitter including excessive Ca^2+^ influx, nitric oxide overproduction, reactive oxygen species formation, decrease of mitochondrial membrane potential, and cellular peroxide formation.

Moreover, HT enhanced the antioxidant defense system by preserving GSH levels and the activities of superoxide dismutase, GSH reductase, and GSH peroxidase [85]. HT alone, or in combination with PUFA (docosahexaenoic and eicosapentaenoic acids), also provided cytoprotection to blood monocyte and neuroblastoma cell lines in a situation of H_2_O_2_-induced oxidative stress. In this case, the cytoprotective effect was evidenced as an increased resistance of cellular DNA damage [86].

An olive mill wastewater extract (whose main constituent is HT, 45.5%) was used to study HT cytoprotective effects in an *in vitro* and *ex vivo* model using dissociated brain cells from mice. Oxidative and nitrosative stress were induced using ferrous iron (a hydroxyl radical generator) and sodium nitroprusside (an NO releaser), respectively. After measuring the mitochondrial membrane potential, the ATP levels, and lipid peroxidation it was concluded that sub-chronic (12 days) administration of HT enhanced the resistance of brain cells to oxidative stress. It is of interest that these findings did not occur with acute HT administration [87]. In a similar, subsequent experiment, the same research group studied the effects of the extract and HT itself in an *in vitro* model of neuronal-like PC12 cells. The results supported their previous observations towards the role of HT as a cytoprotective agent in neuronal-like cells [88].

**Table 1:** *In vitro* and *ex vivo* studies related to hydroxytyrosol (HT) neuroprotective effects via direct antioxidant actions.

Authors and Reference	Experiment Model	Treatment	Nature of the Study	Damaging Agents	Exposure Time	Evaluations	Outcome
Hashimoto *et al.*, 2004 [84]	PC12 cells	HT	*In vitro*	Xantine H_2_O_2_ Fe^2+^	6 and 18 h	- LDH - Catalase - GPx - MTT assay	HT protected the cells against oxidant stimuli via catalase activity
Koo *et al.*, 2006 [85]	Primary cultures of rat cortical cells	HT Caffeic acid Acteoside	*Ex vivo*	Glutamate (100 µM)	1 h (+24 h)	- MTT test - Nitrite and Ca^2+^ - Cellular peroxide - Enzymatic activities - GSH - Lipid peroxidation - MMP assay	Attenuation of glutamate-induced neurotoxicity
Young *et al.*, 2008 [86]	IMR-32 U937 and limphoblastoid cell lines	HT with or without PUFA	*In vitro*	H_2_O_2_	30 min and 6 h (and 24 h)	- Comet assay	Decrease in the level of H_2_O_2_-induced DNA damage
Schaffer *et al.*, 2007 [87]	Dissociated brain cells from NMRI mice	HT-rich olive mill wastewater extract (45.5% of HT)	*In vitro* and *ex vivo*	Ferrous iron Sodium nitroprusside	12 days (subchronic) 100 mg/kg	- MMP assay - ATP assay - Lipid peroxidation	Enhanced cell-resistance to oxidative stress
González-Correa *et al.*, 2008 [89]	Hypoxia-reoxygenation model in rat brain slices	HT and HT acetate	*In vitro* and *ex vivo*	Oxygen and glucose deprivation	7 days 5–10 mg/kg/day p.o.	- LDH assay	Reduction in brain cell death
Schaffer *et al.*, 2010 [88]	PC12 cells	HT-rich olive mill wastewater extract and HT	*In vitro*	Ferrous iron Sodium nitroprusside	18 h	- MTT assay - ATP assay - MMP assay	Brain cell cytoprotection
Muñoz-Marín *et al.*, 2012 [90]	Hypoxia-reoxygenation model in rat brain slices	HT and HT alkyl ether derivatives (C2-C12)	*In vitro* and *ex vivo*	Oxygen and glucose deprivation	7 days	- LDH assay - Lipid peroxidation - GSH - Nitrite and nitrate - 3-nitrotyrosine - Interleukins - MTT assay	Reduction in oxidative and nitrosative stress. Decrease in production of pro-inflammatory interleukins

Abbreviations: GSH: Glutathione; GPx: Glutathione peroxidase; HT: Hydroxytyrosol; LDH: Lactate dehydrogenase; MMP: mitochondrial membrane potential; MTT: 3-(4,5-dimethylthiazol-2-yl)-2,5-diphenyltetrazolium bromide; p.o., oral administration; PUFA: polyunsaturated fatty acids.

Additional evidence of the HT neuroprotective role was found studying the effects of HT and HT-acetate using an *in vitro* experimental model of hypoxia-reoxygenation in rat brain slices. Neuronal damage was evaluated measuring lactate dehydrogenase efflux. Both HT and HT-acetate inhibited the efflux in a concentration-dependent manner. This finding, however, did not occur with other common antioxidants such as vitamin E or *N*-acetylcysteine (lipid peroxidation inhibitors) which suggested that another mechanism, besides the antioxidant, was behind the effects. This HT-mediated neuroprotective effect was confirmed in rats that had been administered with HT and HT-acetate [89]. Recently, the hypoxia-reoxygenation model has been used in rat brain slices to demonstrate that the HT cytoprotective mechanism is mediated by a reduction of oxidative and nitrosative stress, as well as a reduction in the pro-inflammatory interleukin levels [90].

#### 4.4.2. Reversing Specific Damage Present in Neurodegenerative Diseases

*In vitro* studies with olive leaf extract have shown that its main component, oleuropein (Figure 1), has antioxidant protective effects against 6-hydroxydopamine-induced PC12 cell apoptosis. These results suggest that oleuropein may have neuroprotective and neurorestorative properties which could be of interest as a potential treatment of Parkinson’s disease [91].

The potential role of HT, and its precursor oleuropein, as neuroprotective compounds has also been studied *in vitro* in AD models. Neuropathological hallmarks of AD include the accumulation of extracellular neuritic (also named senile or amyloid) plaques (which are formed of amyloid-β peptides) and intracellular neurofibrillary tangles (whose major component is hyperphosphorylated tau protein) [92,93]. In the case of amyloid plaques, their major component, the amyloid-β peptide (Aβ), is between 38 to 42 amino acids in length. Aβ peptides tend to aggregate and form neurotoxic oligomers that trigger pathological cascades of events that lead to cell death [94].

It is noteworthy that HT, oleuropein, and oleuropein aglycone have the ability to prevent tau fibrillization *in vitro* [95]. Oleuropein aglycone also hinders amyloid aggregation of Aβ_1–42_ and its cytotoxicity by preventing the formation of toxic oligomers [96]. The same research group had previously described that, in a similar way, oleuropein aglycone interfered with the aggregation of amylin (a peptide that regulates glycaemia) avoiding the formation of toxic aggregates [97]. In a different study, oleuropein aglycone injected into the rat brain was able to circumvent Aβ toxicity and inflammation [98]. HT and tyrosol were able to protect neuronal cells against Aβ induced toxicity and prevented the nuclear translocation of transcription factor NF-κB [99].

NF-κB is a central regulator of inflammatory activity which activates many genes encoding for pro-inflammatory cytokines and immunoregulatory mediators. Consequently, it has been linked to several major chronic diseases including AD [100]. Additional studies in endothelial cells [101] and in a human monocytic cell line [102] provided more evidence regarding HT ability to reduce NF-κB activation and its translocation to the nucleus [100].

Future *in vitro* studies concerning HT beneficial effects should employ physiological concentrations of this compound. This fact is especially important in the field of neuroprotection, where the concentrations used in the experiments should be realistic and similar to those that could be reached in the brain.

### 4.5. HT Neuroprotective Properties: In Vivo Evidence in Animal Models

Despite the *in vitro* evidence of the potential role of HT as a neuroprotective agent, there is, unfortunately, little *in vivo* research focused on HT itself as a therapeutic target in neurodegenerative diseases (Table 2). In order to be a good candidate as a potential treatment in neurodegenerative diseases, HT should be able to cross the blood-brain barrier and reach the brain. As previously mentioned, the ability of HT to cross this barrier was first described by D’Angelo *et al.* [56]. In these experiments, ^14^C-labelled HT was injected (1.5 mg/kg, i.v.) into rats and later detected in reduced amounts in the brain [56]. Subsequently, another research group detected and quantified free HT in brain microdialysates of anesthetized rats treated with high doses of HT (100 mg/kg, i.v.) [103]. It is worth mentioning that in order to obtain accurate measurements of brain polyphenols exposure, exsanguination and perfusion are required and, if not possible, the values need to be corrected for residual blood in the brain. This is of importance since some phenols found in the brain come from animal experiments that were performed without perfusion [104].

Although the studies evaluating HT neuroprotective effects in animal models are scarce, they provide very useful information in order to evaluate the potential action of this compound in the treatment of neurodegenerative diseases.

In the case of olive oil, extra virgin olive oil (EVOO) was administered to SAMP8 mice for 6 weeks and resulted in beneficial effects towards the learning and memory deficits that characterize this strain of mice. The increase in GSH levels, higher superoxide dismutase and GSH reductase activities, and decreased levels of 4-hydroxynonenal and 3-nitrotyrosine suggested that the effects were mediated by reversing oxidative damage in the brain [105].

The effect of oral administration of EVOO and HT on brain oxidative stress has also been studied in a Huntington disease-like model on Wistar rats. The results show that EVOO and HT reduce lipid peroxidation and increase cellular GSH as a natural mechanism for enhancing protection against oxidative damage by acting as brain antioxidants [106].

In another study, the effects of an eight-week dietary supplementation of oleuropein aglycone (50 mg/kg) were examined in young (1.5 month-old) and middle-aged (4 month-old) transgenic TgCRND8 mice (a model of Aβ deposition). The treatment strongly improved their cognitive performance, reduced brain Aβ levels and plaque deposits, and suggested that the oleuropein aglycone mechanism of action involved the mTOR pathway [107]. The same study and model was extended to aged mice (10 month-old). The results evidenced that the age-dependent deposition of toxic Aβ was reduced in the group whose diet was supplemented with oleuropein aglycone. This reduction was parallel to a reduction in glutaminyl cyclase expression (the enzyme that catalyzes the formation of Aβ peptides), an activation of neuronal autophagy, and an improvement of synaptic plasticity [108].

In a recent study, C57BL/6 mice were injected with oligomeric Aβ_1–42_ plus ibotenic acid to induce neuro-behavioral dysfunction. After treatment, a severe impairment in the visuospatial and working memories took place. However, an HT treatment of 10 mg/kg for 14 days significantly improved spatio-cognitive performances. Further research regarding the neuroprotective mechanisms showed that, in hippocampal neurons, HT treatment was able to reverse the dysregulation of the signaling mechanisms. These include ERK-MAPK/RSK2, PI3K/Akt1, and JAK2/STAT3 survival signaling pathways. Interestingly, the authors also found that HT down-regulated NF-κB [109].

**Table 2:** *In vivo* studies related to hydroxytyrosol (HT) neuroprotective effects.

Authors and Reference	Animal Model	Treatment	Results	Conclusions
Farr *et al.*, 2012 [105]	SAMP8 mice 11 month old	EVOO containing 210 mg/Kg phenolic compounds p.o., 75 µL, 6 weeks	↓ T-Maze retention ↑ Memory in object recognition ↑ GSH levels ↑ GSH reductase activity ↑ SOD activity ↓ 4-HNE and 3-NT	Reversion of learning and memory impairments
Tasset *et al.*, 2011 [106]	Wistar rats (only ♂) 3 months old	(i) Induction of oxidative stress with 3-NP, i.p. 20 mg/kg, 4 days (ii) EVOO and HT (2.5 mg/kg/day, p.o., 14 days	↓ Lipid peroxidation ↑ GSH levels ↑ Succinate dehydrogenase activity	Reversion of oxidative damage induced by 3-NP
Grossi *et al.*, 2013 [107] and Luccarini *et al.*, 2014 [108]	Double transgenic TgCRND8 mice (♂ and ♀) 1.5 month old, 4.5 month old, and 10 month old	Oleuropein aglycone (50 mg/kg of diet) 8 weeks	↑ Cognitive performance ↓ Brain Aβ levels ↓ Aβ aggregation ↓ Plaque deposits ↑ Neuronal autophagy ↑ Histone acetylation	Oleuropein avoids Aβ aggregation and improves synaptic function
Arusundar *et al.*, 2014 [109]	C57BL/6 mice (only ♂) 6–8 weeks old	(i) Induction of neurobehavioral dysfunction with oligomeric Aβ_1–42_ plus ibotenic acid, i.c.v., 1 µL (ii) HT (10 mg/kg/day, p.o., 14 days)	↑ Spatial cognition Stabilization of the dysregulation of survival signaling pathways	HT attenuates the spatio-cognitive deficits induced by oligomeric Aβ_1–42_ plus ibotenic acid
Zheng *et al.*, 2014 [110]	Sprague–Dawley rats (♂ and ♀)	(i) HT 10 and 50 mg/kg/day, p.o., 2 weeks before mating (ii) Exposition to restraint stress (days 14–20 of pregnancy)	↑ Cognitive function of male offspring Modulation of mitochondrial content and phase II enzymes	HT restores learning capacity and memory performance, promoting cognitive function

Abbreviations: EVOO: Extra virgin olive oil; GSH: glutathione; 4-HNE: 4-hydroxynonenal; i.c.v., intracerebroventricular; i.p., intraperitoneal, 3-NP: 3-nitropropionic acid; 3-NT: 3-nitrotyrosine; SOD: Superoxide dismutase; p.o., oral administration.

Finally, it has been recently described that the maternal administration of HT to rats (10 and 50 mg/kg/day, gavage) is able to restore the impaired neurogenesis and cognitive function caused by prenatal stress. In these experiments, exposure to restraint stress during pregnancy led to impairment in learning capacity and memory of the offspring. Analysis of the hippocampus showed that stress induced a down regulation of neural proteins (such as BDNF, GAP43, and synaptophysin), a low expression of glucocorticoid receptor, and a decrease in the antioxidant defense system (including Nrf2, superoxide dismutase, and HO-1). It is of interest that HT treatment could prevent both cognitive impairment and biochemical alterations [110]. Additional *in vivo* studies focused on oleuropein aglycone and its potential effectiveness against AD has recently been reviewed by Casamenti *et al.* [111].

## 5. Clinical Trials

To the best of our knowledge, up to the present date, no clinical trials have been carried out to investigate HT as a therapeutic tool in the secondary prevention of neurodegenerative diseases. We have identified three ongoing clinical trials: (i) a Phase I clinical trial on multiple sclerosis, in which the benefits of different dietary supplements (including HT) are being researched (NCT01381354), (ii) a nutritional intervention study aiming to evaluate HT effects on phase II enzymes in healthy subjects (NCT02273622) and (iii) an interventional pilot study evaluating the capacity of HT to prevent breast cancer in women at high risk of suffering this pathology (NCT02068092).

## 6. Other Olive Oil Minor Components with Important Biological Activities

### 6.1. Triterpenic Acids

**Figure 4:**
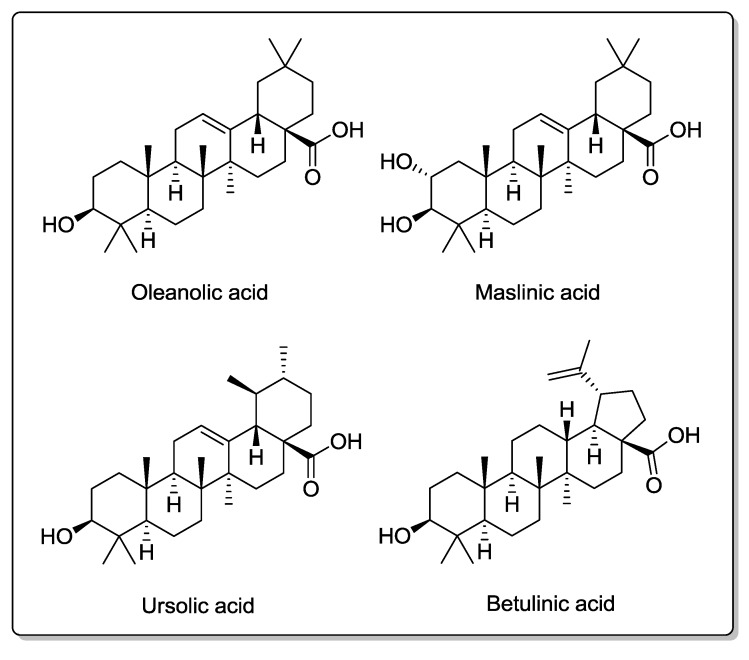
Chemical structures of triterpenic acids present in olive oil.

Oleanolic acid, maslinic acid, ursolic acid, and betulinic acid (Figure 4) are pentacyclic triterpenes that are present in small amounts of olive oil. Several studies have demonstrated that these compounds have anti-inflammatory, antioxidant, antihypertensive, antihyperlipidemic, antidiabetic, antiviral, and antitumoral properties [112]. Recent research suggests that they also have neuroprotective effects. In this sense, maslinic acid neuroprotective effects have been reported in cortical neurons against oxygen–glucose deprivation-induced injury and glutamate toxicity [113,114]. Additionally, it has been described that oleanolic acid protects the brain during hypoxic injury in rats [115] and that ursolic acid protects the brain against ischemic injury in mice via Nrf2 activation [116].

### 6.2. (−)-Oleocanthal

(−)-Oleocanthal (Figure 1) is a phenolic compound found mainly in freshly pressed EVOO. It is responsible for the bitter taste of EVOO and has shown an anti-inflammatory activity via COX inhibition similar to that of ibuprofen [117]. *In vitro* studies have identified this compound as a therapeutic tool for the control of metastatic cancers [118]. Oleocanthal has recently been proposed as a potential neuroprotective agent against AD due to its ability to reduce the polymerization of tau protein [119,120,121], reduce Aβ aggregation [122], and enhance Aβ clearance from the brain [123].

Although these results are of interest, there is a lack of information concerning oleocanthal pharmacokinetics and metabolism, which is needed in order to appropriately evaluate the biological activities that this compound may have in humans.

## 7. Concluding Remarks

Nutrition can contribute to prevent chronic physiopathological conditions including diabetes, cancer, atherosclerosis, and cardiovascular and neurodegenerative diseases [124]. The MD has been associated with a reduced incidence of neurodegenerative diseases and enhanced cognitive performance [4,13,14]. This fact has attracted researchers’ attention and there have been a number of reports trying to identify which components and microconstituents of this diet, including phenolic compounds, are responsible for these beneficial health effects. In this review we have analyzed the current evidence of HT, one of the active compounds present in olive oil that could be, in part, behind the neuroprotective effects attributed to the MD. Some other minor components of olive oil (triterpenes and oleocanthal) have also been briefly considered.

Some caution is required before attributing biological activities to a compound. Recently, a prospective cohort study of almost 800 adults and 9 years of duration concluded that resveratrol levels achieved with diet did not have an influence on health status or mortality risk [125]. This, and other studies have shown that, despite displaying biological effects, resveratrol has no proven human activity [126]. This fact shows how some interesting results that take place in *in vitro* studies and animals, do not always translate in humans and evidences that *in vivo* studies in humans are of utmost importance.

Toxicity studies have shown that HT is a safe compound this is of interest in the context of preparing nutraceuticals with doses higher than the ones compatible with the MD [127,128]. The thorough analysis of the pharmacokinetic properties and safety profile, as well as the *in vitro* and *in vivo* neuroprotective actions, make HT a good potential therapeutic candidate for the prevention of neurodegenerative diseases. However, several considerations should be taken into account: (i) although there is a wide variety of basic research (mainly *in vitro* and less *in vivo*) that demonstrates HT neuroprotective effects, further applied research in humans is needed to verify that the discoveries generated in research laboratories can be translated into practical clinical recommendations; (ii) attributing the beneficial effects of a well-known healthy diet to a single compound is misleading, even if interfering variables are eliminated. It is evident that other components present and lifestyle factors are likely to contribute to the healthy pattern associated with the MD.

Bearing the previous considerations in mind, the scientific knowledge available indicates that HT is one of the most important antioxidants present in olive oil and presents promising potential in the prevention of neurodegenerative disorders. Further research is needed to demonstrate to what extent this compound contributes to presumed neuroprotective effects of olive oil and MD, and to the management of neurodegenerative diseases.

## Abbreviations

Aβ: amyloid-β peptide
AD: Alzheimer Disease
APP: amyloid precursor protein
ALDH2: aldehyde dehydrogenase 2
DOPAL: 3,4-dihydroxyphenylacetaldehyde
DOPAC: 3,4-dihydroxyphenylacetic acid
DOPET: 3,4-dihydroxyphenylethanol
EVOO: extra virgin olive oil
GSH: Glutathione
GST: Glutathione *S*-transferase
GCL: γ-Glutamate cysteine ligase
HO-1: Heme oxygenase-1
HT: Hydroxytyrosol
JNK: c-Jun N-terminal Kinase
Keap1: Kelch-like ECH-associated protein 1
LDL: low-density lipoprotein
MAO: Monoaminooxidase
MD: Mediterranean diet
MUFA: monounsaturated fatty acids
NQO-1: NAD(P)H: quinone oxidoreductase-1
Nrf2: nuclear factor E2-related factor 2
PON: Paraoxonase
PUFA: polyunsaturated fatty acids
SAMP8: senescence-accelerated mouse P8
